# The Isokinetic Rugby Union Physical Work Evaluation (RUPWE) protocol: Can Rugby Union Players meet the physical work demands of the game?

**DOI:** 10.17159/2078-516X/2021/v33i1a8686

**Published:** 2021-02-24

**Authors:** JT Doran, R Naidoo

**Affiliations:** 1 Just Kinetics Preventive Health Care Provider, Station Road, Carrigans, Lifford, Co-Donegal, F93VYA0, Republic of Ireland; 2 Discipline of Biokinetics, Exercise and Leisure Sciences, College of Health Sciences, University of KwaZulu-Natal, South Africa

**Keywords:** isokinetic, trunk extensors, trunk flexors, mechanical load, spinal injuries

## Abstract

**Background:**

To protect the lumbar spine from excessive forces, rugby union players need to demonstrate the work ability of the trunk extensors and flexors to meet the physical demands.

**Aim:**

To measure and evaluate whether rugby union players were able to meet the imposed physical work demand, considering limitations, tolerances and resistance to fatigue, using isokinetic dynamometry for trunk extensors (TE) and trunk flexors (TF).

**Methods:**

Fifty-five male players, between the ages of 18 and 23 years, participated in the study. All participants completed a PAR-Q (pre-activity risk) questionnaire before the isokinetic testing. Their height was between 1.80 ± 0.67 m and body mass was 86.0 ± 17.5 kg. Participants were subjected to a newly designed protocol using the Biodex Isokinetic System 3 Dynamometer, called the Rugby Union Physical Work Evaluation (RUPWE).

**Results:**

There was a significant difference between the forwards’ trunk extensor peak torque to body weight 488% ± 119% and the trunk flexor peak torque to body weight 289% ± 73%. Furthermore, there was a large effect size between trunk extensor and trunk flexor muscle performance for the forwards (*d* =2.0) and backs (*d* =1.9) for peak torque to body weight. Spearman’s rank-order correlations (r_s_) showed a moderate negative correlation for the forwards between trunk extensor peak torque to body weight and time to peak torque, (r_s_ = −0.4; p=0.018). There is a strong negative correlation for the backs between trunk extensor peak torque to body weight and time to peak torque, (r_s_ = −0.6; p=0.003).

**Conclusion:**

The physical work evaluation protocol can be used as a screening tool for rugby players as it measures the extensive mechanical load placed on the lumbar region. This has the potential to evaluate their athletic performance for the demands of tackling and scrumming.

Current occupational health and safety legislation requires the professional rugby union to comply with the mandate to protect employees against occupational diseases and injuries due to workplace hazards and risks, including ergonomic/human risk factors.^[1]^ Rugby union-related incidence and risk of injury varies from acute catastrophic/traumatic to chronic degenerative, and severity is dependent on playing position, location, magnitude/load and mechanism of injury.^[2–13]^ The incidence of catastrophic spinal injuries and traumatic brain injuries, such as concussion and chronic traumatic encephalopathy, are potentially hazardous to a player’s career and livelihood in rugby union. The performance of the trunk muscles are not only important to athletic performance in rugby union, but in the prevention of catastrophic injuries to the spine and traumatic brain injuries as experienced on head impact at engagement of the scrum, the collapsing or “popping out” of the scrum or tackle.^[2]^ The evaluation of physical performance’s ability against matching imposed physical demands provides a systematic evidence base to assess match readiness and to modify player development pathways to efficient and safe participation in competitive rugby. Professional and novice players of rugby union require a high level of skill, physical tolerance and resistance to fatigue to repeatedly, safely and effectively engage in the match.^[2]^ It is important to understand the physical demands of rugby union^[2]^ with its various implications for coaching, sports medicine, vocational training and occupational health and safety. Research limitations in measuring magnitude/load include inconsistency in the units of measurement with little or no consensus in conformity, biomechanical studies attempting to reproduce the aggressive and physical nature of rugby union, and failure to match the imposed physical demands.^[2–6, 15, 18, 20–23]^

The function of the trunk muscle extensors and flexors are to perform and maintain the position of the joints or body posture, provide joint stability, body manoeuvring, locomotive movements through isolated or coupled muscle performance, and to protect the spine against lumbar mechanical stress from excessive forces. Significant muscle activity is required to constrain spinal motion in an oppressing environment.^[3]^ Muscle balance implies a harmony and synergy between different muscles all working towards the common goal of efficient, effective functional movement. The game of rugby union is dynamic and involves players constantly making postural changes in the match to adapt to game situations. This influences the activation patterns of the trunk muscles in various ways and is dependent upon whether the players are positioned upright, crouching or bending forward in a ruck, maul or scrum, tackling, their speed of movement, and gravity. The trunk extensors (TE) and trunk flexors (TF) function during scrummaging is to oppose impact forces, sustain forward movement, maintain and adjust body position and prevent excessive forward flexion or extension, where the scrum will either collapse or “pop out”. Hendricks^[2]^ identified that between 2008–2012, the scrum phase accounted for 33% of all catastrophic injuries, reporting 58% as occurring on impact at engagement, 37% from a collapsed scrum and 5% from “popping out”. The acute and chronic front row spinal injury burden of the scrum by region is presented as occurring in the cervical (41%), thoracic (56%) and lumbar regions (71%).^[4]^ While it is acknowledged that the risk of injury is multifactorial^[3,6–8]^, the biomechanical or ergonomic demands of rugby union are unique and are likely to significantly contribute to the risk of injury and/or the recurrence of injuries.^[2]^ There has been a proportional increase in the severity of injury and days absent or days lost^[7,8]^ experienced in rugby union, particularly in concussion.

Studies have shown the enormous mechanical loads imposed upon the lumbar region in rugby union^[2–5]^, hence indicating that trunk muscle performance is undeniably important for athletic performance. To the best of the authors’ knowledge, studies show limited consistency across the measures and units of measurement, nor has any study investigated the involvement of both the trunk extensors and flexors in supporting the spine as co-activators and their combined role in muscle performance in rugby union. While the research does present many studies where electromyography (EMG) and maximum voluntary contractions (MVC) are used, which are isometric in nature, very few studies are able to demonstrate a practical evaluation that may be reproduced in exercise, monitored consistently, whilst maintaining validity, reliability and repeatability.^[2–6,12,13,19–22]^

Isokinetic exercise and testing is performed at a pre-set fixed speed allowing for the accommodation of resistance that is equal to the effort applied. The set lever arm’s speed allows the dynamometer to measure the torque output by the participant throughout the range of motion (ROM). This is unlike manual muscle or isometric testing, which are static tests, and isotonic testing which measures strength at the strongest section of the ROM and is also affected by speed of movement. Isokinetic testing provides a safe, objective and reliable evidence base for evaluating muscle performance and determining athletic ability.^[9–11]^

Few studies address the differences between forwards and backs in the evaluation of trunk strength and biomechanical studies have focused on either the tackle or the scrum.^[2–6,12,13,15,19–22]^ Thus far, the authors believe that no study has addressed the muscle performance requirements of both the trunk extensors and flexors for the task demands of these two very important tasks to the game of rugby union, and their application to both the forwards and backs at engagement on impact collisions. The need exists to provide rugby union with a gold standard for the evaluation of muscle performance of the trunk flexors and extensors as it applies to the game of rugby union in limiting head impact exposure, catastrophic spinal injuries, and traumatic brain injuries, such as concussion. A further limitation is that most studies involve only a few participants and are focused mainly on the front row or forwards using scrum simulators.^[5,12,19–22]^ Although some studies attempt to simulate match conditions, they fail to reproduce the load pattern that is as aggressive and physical in nature as a competitive match.^[3]^ Ramesh et al. ^[3]^ maintain that spinal moments vary across populations and are dependent on the participant and the scrum.

Based on the corroboration in the various studies, work ability must be established to measure, at least the very minimum of physical demands that are able to be imposed, while also measuring the participants’ performance abilities, tolerances, limitations and resistance to fatigue^[2–5]^. Using a scrum simulator, Milburn^[12]^ found that the physical demand of the engagement phase of the scrum is characterised by a large forward force which lasts approximately one second and acts to stop opposing forward motions. This may be followed by a decrease in force due to the compression of the body tissues and reorientation of body positions. High forces/impulses ranging from 3 740 N for novices and 7 980 N for international players were found, proposing that these were due to their pronounced weight and the high speeds involved, and therefore assumed that this is due mainly to the momentum generated by speed of engagement rather than active muscle action on impact. The average forward impulse per scrum event, being opposed on impact, may range between three to five times the average body mass as determined in the Milburn^[12]^ study. Therefore, to maintain a neutral spine in order to protect the spine from excessive compressive forces and oppose forward momentum on scrum engagement, the physical demand placed on the TE and TF would require an instantaneous activation of these muscles, to produce an opposing force equivalent to between three to five times the player’s own body mass which would then need to be sustained. A maximum voluntary contraction relative to body mass is an important indicator of performance ability for the evaluation of TE and TF muscle performance during the demand of the scrum.^[12]^

The voluntary muscle contraction of the TE and TF would need to demonstrate the minimum ability to meet this physical demand of 300% relative to body mass. A further indicator of TE and TF muscle performance^[12]^ is the instantaneous active muscle action measured in the time taken to attain the maximum voluntary contraction, by measuring the rate of force development. The muscle performance of the TE and TF would need to demonstrate the minimum ability to meet this physical demand by attaining the maximum voluntary contraction in <1 000 ms. It is important for the TE and TF to instantaneously activate to protect the lumbar spine and the rest of the body from these excessive forces experienced on impact collision during the scrum’s engagement and similarly, in contact during the tackle. In a biomechanical study, Hendricks^[2]^ determined the energy/work load generated on magnitude of impact by front on tackles as being 902 J – 7 608 J and the side-on tackle being 595 J – 6 209 J on impact. Taking into consideration the need to maintain a neutral spine, the high forces and speed on scrum engagement and the subsequent injuries are due to a lack of optimal muscular balance. Therefore, an isokinetic protocol, which is speed and posture/position-dependent, will be able to adequately measure muscle performance, postural balance and stability control by dynamically loading the muscles/joints through their functional range.

Isokinetic testing is able to provide an insightful, objective, evidentiary basis for assessments and therapeutic interventions through these assessments, and reporting and monitoring that is safe, reliable, valid, practical, meaningful and predictive^[9–11]^. The use of isokinetic testing as a modality in the evaluation of the ability of physical performance against matched physical demands provides a systematic evidence-base to assess match readiness and modify player development and rehabilitation pathways. Professional and novice rugby players require a high level of skill, physical tolerance and resistance to fatigue to repeatedly, safely and effectively engage in the demands of the match.^[2]^ The need to measure and evaluate whether rugby union players are able to meet the imposed physical work demand, is warranted. Therefore, this study aimed to measure and evaluate whether rugby union players were able to meet the game’s imposed physical work demand, considering limitations, tolerances and resistance to fatigue, using isokinetic dynamometry for TE and TF.

## Methods

### Study design

This study has a descriptive, cross-sectional design. Permission to conduct the study was obtained from the Sharks Rugby Academy, KwaZulu-Natal, South Africa, and ethical clearance was granted from the university’s Biomedical Research Ethics Committee (BFC337/19).

### Participants

The study was conducted at the Sharks Rugby Academy in Durban, KwaZulu-Natal. Fifty-five players were voluntarily recruited to participate in the study. All participants adhered to the following inclusion criteria: injury free, with no acute injury to the spine/lower back for the six weeks previous to the study; non- symptomatic for lower back pain; healthy with no pre-existing chronic diseases, such as diabetes, heart disease, or injuries. Players were excluded if they were currently undergoing rehabilitation for any lumbar injury, or were found to be hypertensive.

### Test procedures and protocol

**Fig. 1:**
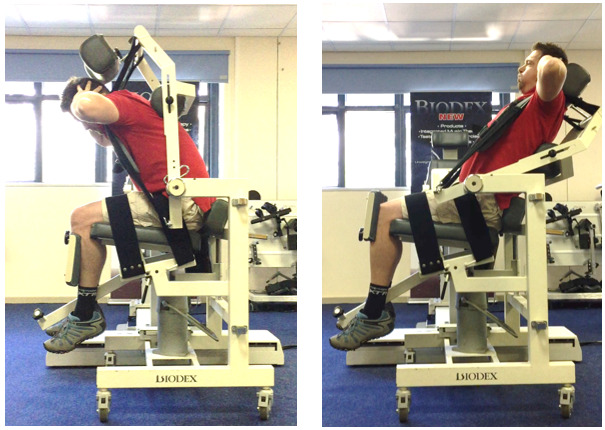
The RUPWE protocol setup

An information session was conducted at the Sharks Rugby Academy prior to testing. The study aim and Rugby Union Physical Work Evaluation (RUPWE) protocol setup (Fig. 1) were explained in detail to the participants, informing them of all risks and benefits associated with their participation. Informed consent was thereafter obtained from all participants.

**Fig. 2:**
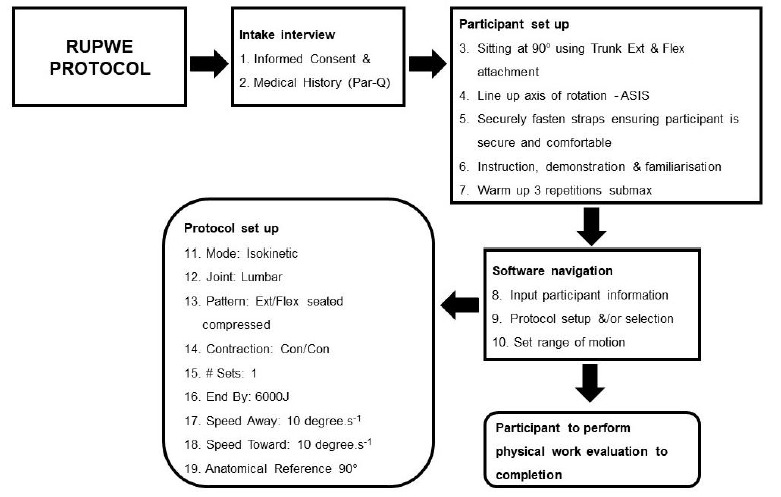
The RUPWE protocol

Fifty-five participants completed the PAR-Q and were all eligible to participate in the study. Thereafter, height and weight were recorded. Participants were instructed on the technique required for trunk extension and flexion. Prior to beginning the evaluation, isokinetic machine/familiarisation was conducted. Using the Biodex Isokinetic System 3 and dual position back attachment, each participant performed the exercise in isokinetic mode, testing the lumbar joint performing extension and flexion. Contraction type was set as concentric and speed was set at 10 degrees.s^−1^ for a single bout of repeated trunk extension and flexion, ending once 6 000 joules of work had been completed (Fig. 2).

### Instrumentation

Height was measured in metres (m) using a stadiometer.^[14]^ Weight (kg) was measured using a calibrated scale with a beam and moveable weights.^14]^ Isokinetic concentric trunk extension and flexion were measured using a Biodex System 3 PRO isokinetic system (Biodex Medical Systems, Inc., New York, USA). The isokinetic system was calibrated before testing. The participants were requested to be seated at 90 degrees and a range of motion for trunk flexion and extension was set at 70 degrees. Axis of rotation used the anterior superior iliac spine. Results were measured in newton metres (N·m).^[10]^ The Biodex Isokinetic System 3 PRO is a reliable and valid method for measuring muscle torque recorded during isolated joint exercise when angular velocity remains constant.^[9]^ Isokinetic dynamometers provide an accommodating resistance equal and opposite to the muscular forces applied for maximum external force output with changing positions of the joint.

The data collection was conducted by a biokineticist, trained in using the isokinetic device. The data were collected in a controlled environment, entered directly into the computer and data station of the isokinetic system.

### Data management

The forwards were grouped together as playing positions 1–8 and the backs were grouped together as playing positions 9–15. Each participant was allocated a unique code. Their codes were entered into the isokinetic computer software system prior to the start of the testing protocol. Data were then exported into an Excel spreadsheet for statistical analysis.

### Statistical analysis

All the data were analysed using the Statistical Package for the Social Sciences, version 21. Data were quantified using descriptive statistics through means and standard deviations, and the distribution comparing the descriptive results used the Wilcoxon signed ranks test and a Mann-Whitney U-test to investigate the differences between extensors and flexors among the forwards and backs. A binomial test was used to investigate the proportion to which the extensors and flexors are able to meet the physical demands of the game. Additionally, Cohen's *d* was used to describe the standardised mean difference of an effect. A Spearman’s Rho (r_s_) correlation analysis was used to investigate the difference between flexors and extensors among forwards and backs. The level of statistical significance was set at p < 0.05.

## Results

Fifty-five rugby union participants, aged between 18 and 23 years, were tested; 29 (53%) were forwards and 26 (47%) were backs. Their mean height was 1.80 ± 0.67 m and mean body mass was 86.0 ± 17.5 kg).

The results for the forwards and backs are presented separately to identify and compare the differences in trunk extension and flexion muscle performance between them. The majority of the tests between the forwards and backs yielded significant differences. Mean isokinetic differences are presented in Table 1.

There were no significant differences in the number of repetitions completed (the mean was 16 ± 4 repetitions); in the range of motion (the mean was 71.6 ± 2.1°); or in the agonist/antagonist ratio, (the mean agonist/antagonist ratio was 63% ± 12% for TE and TF between the forwards and the backs). Additionally, there were no significant differences for acceleration (the mean TE acceleration time was 142 ± 159 ms and mean TF acceleration time was 169 ± 188 ms). There was also no significant difference for deceleration time. The mean TE deceleration time was 3 039 ± 3 000 ms and mean TF deceleration time was 2273 ± 2 757 ms.

Using a binomial test, the TE demonstrated a significant 96% ability to match the required minimum physical demands, with p < 0.0005 for Peak Torque to Body Weight (%) (PT/BW) > 300%. However, the TF muscles showed no significant difference in proportion to the matching ability required to meet the minimum physical demands of PT/BW % > 300%.

The TF muscles had a significant 82% ability to match the required minimum physical demands of time to peak torque (TPT) < 1 000 milliseconds (ms), p < 0.0005. There was a moderate negative correlation for the forwards between trunk extensor PT/BW and TPT, ((r_s_ = −0.4), p = 0.02). There was a strong negative correlation for the backs between trunk extensor PT/BW and TPT, ((r_s_ = −0.6), p = 0.003) (Table 2).

## Discussion

The study investigated whether the isokinetic TE and TF protocol of work ability measures and evaluates rugby union players’ ability to meet the physical work demands for tolerances, limitations and resistance to fatigue imposed by the sport. The slow speed of the protocol at 10 degrees.s^−1^ produces a load pattern that is both physical and aggressive to simulate the nature of a competitive match. A 90° - 90° scrum position is adopted, to isolate the TE and TF and minimise force coupling in multi-joint muscle activation and to further increase compressive forces the evaluation is conducted in a seated position. The baseline for the protocol development was created by identifying a minimum work load pattern as observed by Hendricks^[ 2]^ and used the magnitude of the side-on tackle to simulate a maximum work load set at 6 000 J. Hendricks^[ 2]^ suggests a theoretical model for the relationship between the number of tackles in which a player engages in acute and chronic fatigue, magnitude of impact (energy load), markers of muscle damage (microtrauma) and how this relationship interacts with the tackle injury risk (tolerance overload and reduction) and tackle performance. Quarrie and Hopkins^[13]^ determined the number of tackles in a match may range from 10 to 35 tackles and may be dependent on playing position. The protocol allowed for repeated postural change for the duration of the evaluation of forward bending and backward extending, producing dynamic muscle activity and placing extensive mechanical load on the lumbar region.

The mean performance of the forwards and the backs for the TE demonstrated a significant ability to match the imposed physical demands of work ability for PT/BW. This seems to support the results found in Cazzola et al.^[18]^ which identify the preactivation of the erector spinae (ES) prior to scrum engagement being >25% MVC; however, in the Cazzola study the ES significantly reduces during the engagement and sustained phase for the crouch-bind-set (CBS) and crouch-touch-set (CTS) procedures of scrum engagement. This could support the Milburn^[12]^ study, noting muscle tissue compression and reorientation of body position allowing for compensatory muscle activation, such as the eccentric activation of the TF. The mean performance of the TF for the forwards demonstrated a significant inability to match the imposed physical demands for PT/BW. This may indicate a limitation in the TF to tolerate the normal load of excess energy transferred on the impact of engagement in a single contact of concentric muscle activation.^[2–6,13,15]^ The consequence of this finding supports the potential catastrophic injuries sustained on impact of engagement and the collapsing of the scrum as detected in the Hendricks^[2]^ study. However, this could more specifically indicate the limitations in the eccentric muscle activation of the TF during compression of the opposing impact on engagement and the downward forces experienced during the sustained push and reorientation of body positions and the effect of gravity. Improving the eccentric strength of the TF could assist in preventing the scrum from collapsing and players from developing catastrophic spinal injuries, traumatic brain injuries or sports-related concussion. This TE/TF dynamic muscle strength imbalance suggests that there may be a reduced physiological capacity in the function of the TF to harmoniously synchronise with the TE to resist distortion of the muscles and joints when experiencing forces of equal or greater magnitudes, such as observed on the impact of the scrum or impact collisions on contact during the tackle.^[2–6, 13,15,16,18]^ Muscle balance implies a harmony and synergy between different muscles, all working towards the common goal of efficient, effective functional movement, which would require concentric and eccentric coactivation of the agonist and antagonist during joint movements. Isokinetic eccentric exercise is a practical, safe, effective and efficient way to isolate and condition the TF which would improve the muscle performance to provide the necessary dynamic support as required during the scrum.

The higher PT/BW observed for the TE may be due to the biomechanical linkages in the skeletal system that, through strain transmission, actively engages proportionally more of the myofascial chain.^[15]^ These authors believe that this may also be as a result of motor pathway development patterns that have been established through traditional methods in coaching, strength and conditioning exercises, such as the deadlift, and specificity of training patterns during coaching sessions, e.g. scrummaging against a scrum machine as observed in the differences of the TF between the forwards and the backs. Further investigation into the myofascial chains, combined with traditional strength and conditioning exercises and exacerbated by the specificity of set coaching practices, are more likely to explain the effectiveness of PT/BW development in the TE as opposed to the TF. Therefore, it is reasonable to assume that the volume of strain transmission and cumulative loading of the mechanoreceptors have contributed to the multifactorial physiological adaptation of the TE, as observed in the results of this study. The rationale lends itself to a positive adaptation of the physically imposed demands of training observed in the TE PT and PT/BW.

As the participants of the study were injury-free, the strain transmission experienced by the TF in both the forwards and the backs seems to indicate that the loads experienced in strength and conditioning training, or during practice sessions, fails to optimally load the TF.^[12]^ The rationale lends itself to a negative adaptation to physically imposed demands during training. It seems that traditional strength and conditioning/coaching methods are ineffective in matching the dynamic strength produced by the TE for the TF.

**Fig. 3:**
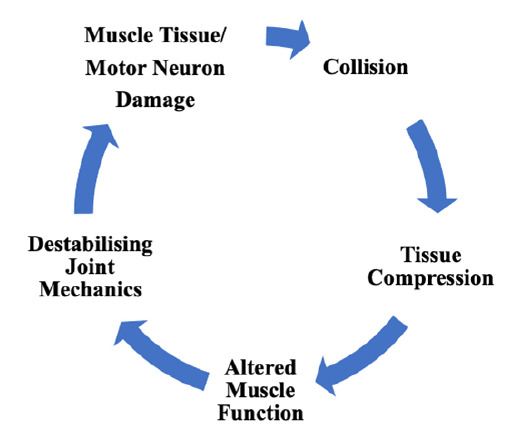
Theoretical model describing the aetiology of repetitive collisions on contact in rugby union

The rate of force development in the TE muscle activation sequence for both the forwards and the backs is significantly slower when compared with TF muscle activation. There is a significant inability of the TE in both the forwards and the backs to meet the required imposed physical demands for work ability for TPT. This inability, as demonstrated, to match the imposed physical demands for TPT, also indicates an inability to rapidly activate the muscle to produce the maximum dynamic force needed to maintain the structural tensegrity required on impact to tolerate the normal load of excess energy transferred on a single contact.^[2–6, 15]^ Impact on engagement is dependent on speed and mass; and in rugby union, the magnitude of impact on engagement has been established as being high.^[2–6,15]^ The player's musculoskeletal system must respond rapidly to provide the necessary structural tensegrity to tolerate the impact experienced on contact engagement. In an unpublished study by Pennington^[24]^ investigating neck strength and head acceleration in rugby union, the author demonstrated a correlation between neck muscle strength and head acceleration and determined that neck strength significantly slowed down head acceleration on contact in tackle engagements reducing the risk of concussion and traumatic brain injury. Similarly, strengthening the TE and TF to respond rapidly to produce maximum voluntary contraction on impact may reduce body and head accelerations minimising the risk of developing catastrophic spinal injuries and or traumatic brain injuries, such as concussion. In this study, both the TE and TF demonstrated a deceleration time >1 000 ms. The mean TE deceleration time was 3 039 ± 3 000 ms and mean TF deceleration time was 2 273 ± 2 757 ms. The inability to rapidly respond to the opposing impact forces may result in destabilising joint mechanics, altered muscle function, and tissue compression to the point of microtrauma, resulting in tissue and/or motor neuron damage.^[2–6,15]^ Fig. 3 illustrates a proposed theoretical model of the compounding effect of macro- or microtrauma for repetitive collisions on contact. The results maintain the importance of the rate of force development in TE muscle activation in supporting and maintaining trunk stability against posterior and coactivation of TF against anterior lumbar mechanical stress perturbations in rugby union players. The results of the slow rate of force development as demonstrated in both the forwards and the backs may be evidence that the TE is predominantly tonic in constitution, and the TF may be more phasic in their constitution. Regardless, careful manipulation of exercise could result in adaptation to the TE motor neuron, or even conversion of fibre type 1 to fibre type 2.^[19]^ Isokinetic exercise at high velocity and in both concentric and eccentric muscle activation patterns would condition the TE and TF towards an improved rate of force developments and improve the neuromuscular control of these muscles.

The literature on the effects of fatigue in rugby performance seem to present conflicting results.^[20–23]^ The RUPWE protocol’s results in this study indicate that the TF is significantly more prone to fatigue, as work fatigue is >25% in both the forwards and backs. Though the development of muscle fatigue in this study may not be a reflection of how muscle fatigue manifests during rugby, it does demonstrate significant muscle fatigue experienced during the workload as determined to occur in a single side-on tackle. The TE may be more tolerant of, and resistant to, fatigue ^[20–23]^ than the TF. The relationship between total work for the TE and TF could be improved by careful manipulation of exercise and could result in the adaptation to the motor neurons or muscle type conversion.^[19]^ The relationships between the TE and TF as investigated in this study should be central when developing training and or rehabilitation programmes for return to play.

## Conclusion

The RUPWE protocol determines whether the TE and TF are able to meet the physical demands imposed on them by rugby union. Rugby union can now use the protocol described in this study as a screening tool that has the ability to safely and objectively predict for injury and improve rehabilitation pathways as it measures the extensive mechanical load placed on the lumbar region. The use of the protocol in combination with the eccentric exercise of the TF could reduce the risk of the scrum collapsing, thereby minimising the incidence of head impact exposure which could result in catastrophic spinal injuries and traumatic brain injuries in sports-related concussion. The protocol establishes a baseline of work ability equivalent to the magnitude or load of a single side-on tackle. Therefore, it may be used to identify the limitations in tackle performance by reducing tackle injury risk when considering the number of tackles made in a match. Thus the protocol may be used for injury prevention and for evaluating athletic performance in rugby union players. The use of isokinetic exercise in conjunction with the RUPWE protocol may assist coaches and medical staff in identifying and rectifying loading conditions to optimally rehabilitate and develop load progressions for return to play and athletic performance development pathways. A coordinated approach to strengthen and prepare a team for all possible scenarios is required.

## Figures and Tables

**Table 1:** Mean trunk extension and flexion isokinetic results between forwards and backs

Variable	Forwards (n=29)				Backs (n=26)			

	Trunk Extension	Trunk Flexion	Cohen’s *d*	p value	Trunk Extension	Trunk Flexion	Cohen’s *d*	p value
Peak Torque (N·m)	452 ± 128	270 ± 128	1.7	< 0.0005	365 ± 72	237 ± 47	2.1	< 0.0005
Peak Torque to Body Weight (%)	488 ± 119	289 ± 73	2.0	< 0.0005	487 ± 115	313 ± 61	1.9	< 0.0005
Time to Peak Torque (ms)	1 411 ± 1 525	772 ± 1 325	0.5	< 0.0005	1 518 ± 1 575	560 ± 642	0.8	< 0.0003
Work Fatigue (%)	18 ± 13	29 ±13	2.7	< 0.0005	16 ± 14	33 ± 12	3.0	< 0.0005
Total Work (Joules)	4 208 ± 211	1 733 ± 198	−0.1	< 0.0005	4 107 ± 269	1 821 ± 281	−0.5	< 0.0005
Position of Peak Torque (°)	82 ± 19	35 ± 16	12.1	< 0.0005	79 ± 19	33 ± 10	8.3	< 0.0005
Coefficient of Variance (%)	15 ± 8	16 ± 7	−0.9	-	16 ± 8	19 ± 8	−1.3	0.028

Where applicable data are expressed as mean ± SD

**Table 2:** Spearman’s rho (r_s_) between peak torque to body weight (%) and time to peak torque (ms)

Position	Trunk Extension r_s_	p value	Trunk Flexion r_s_	p value
Forwards (n=29)	−0.4	0.018*	−0.5	0.013*
Backs (n=26)	−0.6	0.003**	−0.3	0.197

* Correlation is significant at the 0.05 level (2-tailed)

** Correlation is significant at the 0.01 level (2-tailed)
